# *Tian Jiu* therapy for allergic rhinitis: study protocol for a randomized controlled trial

**DOI:** 10.1186/s13063-016-1374-5

**Published:** 2016-05-17

**Authors:** Wai Kun, Linda L. D. Zhong, Liang Dai, Chung-Wah Cheng, Ai-Ping Lu, Zhao-Xiang Bian

**Affiliations:** School of Chinese Medicine, Hong Kong Baptist University, 7 Baptist University Road, Kowloon Tong, Hong Kong, China; Hong Kong Chinese Medicine Clinical study Center, Hong Kong Baptist University, 205, Jockey Club School of Chinese Medicine Building, 7 Baptist University Road, Kowloon Tong, Kowloon, Hong Kong

**Keywords:** Allergic rhinitis, Chinese herbal medicine, *Tian Jiu*, Randomized controlled trial

## Abstract

**Background:**

Allergic rhinitis (AR) is one of the most common allergic diseases. The conventional treatments of allergic rhinitis are oral anti-histamines, the use of intranasal corticosteroids, and immunotherapy. Dissatisfied with the ineffectiveness and side effects of these treatments, substantial numbers of patients are turning to alternative treatments like Chinese herbal medicine, particularly *Tian Jiu* (TJ). TJ is a form of moxibustion in which herbal patches are applied to specific acupoints on the skin. This study aims to investigate the efficacy and safety of TJ in the treatment of allergic rhinitis.

**Methods/design:**

This will be a prospective, randomized, single-blinded, controlled trial in patients with AR. After a 1-week run-in period, eligible subjects will be randomly assigned to the TJ group, placebo-control group or waitlist-control group. The TJ and placebo-control groups will undergo a 4-week treatment with one session per week and one 4-week post-treatment follow-up. Participants in the waitlist-control group will not receive any treatment during the first 4 weeks but will be required to be assessed. The primary outcome will be the change in the weekly average of the Total Nasal Symptom Score recorded from baseline to the end of treatment. The secondary outcomes will be change in symptoms and change in need for medication between baseline and the end of treatment by using the Rhinitis Quality of Life Questionnaire. Rescue medication (RM) needs will be measured using an RM score, comprising the weekly sum of daily assessments and any form of systemic steroids for allergic rhinitis.

**Discussion:**

This study will be the first study to compare TJ treatment for allergic rhinitis with a placebo-control group, and a waitlist-control group. The investigation of TJ for allergic rhinitis will also suggest recommendations for clinical practice. The results of this study are expected to provide consolidated evidence for the effectiveness and safety of TJ for the treatment of patients with allergic rhinitis.

**Trial registration:**

NCT02470845 (17 May 2015).

## Background

Allergic rhinitis (AR), which can be acute or chronic, is one of the most common allergic diseases. A number of surveys have documented the high prevalence of AR and the substantial burden to patients and the health care system [1–3]. In the United States, the prevalence of AR was shown to vary from 10 to 30 % [4]. In Europe, the prevalence was around 23 % [5]. In 11 cities in China, the prevalence of AR was shown to vary from 8 to 24.1 % [6]. The conventional treatment of AR symptoms, such as nasal obstruction, rhinorrhea, sneezing, and itching, includes the use of intranasal corticosteroids, oral anti-histamines with or without decongestants, immunotherapy, and education [7]. Substantial numbers of patients with AR are dissatisfied with conventional medical treatment and repeatedly experience side effects. As a result, many AR sufferers are turning to complementary and alternative treatments [8, 9]. Among them, Chinese medicine, including herbal medicine, acupuncture and *Tian Jiu* (TJ), are frequently used to manage AR in East Asia [10].

In China, traditional Chinese medicine (TCM) has a long history of using TJ as a measure to prevent asthmatic attacks [11]. TJ as an external application is also known as “drug moxibustion” or “vesiculating moxibustion.” Herbal patches are applied on the selected acupoints or the diseased part. In TCM, this treatment regulates the functions of channels and *zang-fu* organs. It can warm the channels and disperse coldness. It is also able to invigorate *qi* movement, harmonize nutrient absorption and defense mechanisms, and resolve stagnation in the body and stasis of the blood. The mechanisms of TJ, which have been studied in recent years, show that it can inhibit the release of mast cell mediators and modulate serum IgE levels, lymphocyte and/or macrophage activity [12].

Currently, some clinical studies have showed that TJ therapy is effective in treating AR. They have found that TJ therapy is better than placebo for treating clinical symptoms and signs as well as improving quality of life [13, 14]. One previous study has also shown that the TJ group had lower scores for sneezing, rhinorrhea and nasal stuffiness after finishing therapy when compared with the control group and also showed significant improvement in the scores for the 36-item Short Form Health Survey (SF-36), which measures patients’ quality of life [15]. A study using the Rhinitis Quality of Life Questionnaire (RQLQ) to assess quality of life also demonstrated that TJ therapy can improve quality of life when compared with a placebo group [16].

In recent years, clinical and basic researchers have found that TJ acts as a modulator of anti-inflammatory cytokines [17] and affects neuro-immunological mechanisms, e.g., lowering plasma levels of vasoactive intestinal peptides and substance P [18]. However, these studies were done on a relatively small scale and did not use a randomized control design.

In order to further investigate the efficacy of TJ in the treatment of AR, a rigorous randomized controlled trial will be conducted in our clinical centers. The evidence from this rigorous clinical research, if successful, will become the basis for a large multi-center study. The overall goal is to develop evidence-based clinical guidelines for the use of TJ therapy in treating AR.

The research was supported by the Marcoda Co. Ltd. Research Fund and was registered with the identifier NCT02470845 (17 May 2015) at ClinicalTrials.gov. The funding agency had no role in the development of the study design, data collection, or manuscript preparation for publication.

### Objective

The aim of this study is to investigate the efficacy of TJ in the treatment of AR, by comparing it with a placebo-control group and a waitlist-control group, in Hong Kong.

## Methods/design

### Study design

This will be a prospective, randomized, single-blinded, controlled trial in patients with AR. After a 1-week run-in period, eligible subjects will be randomly assigned to the TJ group, placebo-control group or waitlist-control group. Except for rescue medication (RM) drugs, no other routine medication use for AR symptom control will be permitted in the entire study.

**Fig 1:**
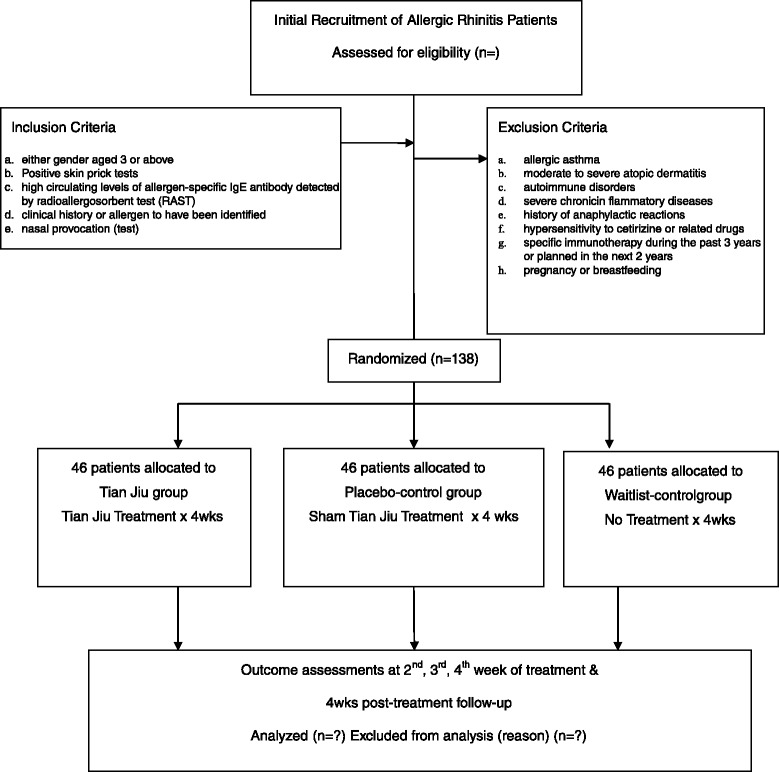
Participant flowchart

**Fig 2:**
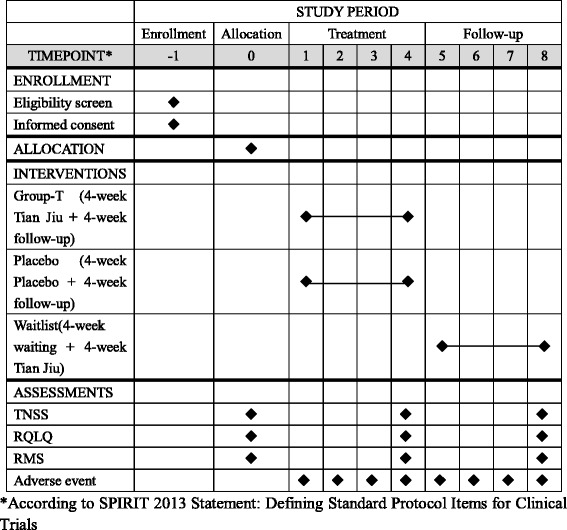
Schedule of enrollment, intervention and assessment

The TJ group will undergo a 4-week treatment with herbal patches for one session per week, and a 4-week post-treatment follow-up of one assessment session per week. The placebo-control group will undergo a 4-week treatment with placebo patches for one session per week, and a 4-week post-treatment follow-up of one assessment session per week. The waitlist-control group will receive no treatment during the first 4 weeks but, beginning at the fifth week, this group will receive TJ treatment for 4 weeks as compensatory. All groups will be assessed in the same way throughout the study period. The total study period will be 9 weeks. The participant flowchart is listed in Fig. 1 and participant timeline is listed in Fig. 2.

The study protocol has been approved by the Hong Kong Baptist University Ethics Committee on the Use of Human Subjects for Teaching and Research (Approval number HASC/13-14/0241), and informed consent was obtained from each participant.

### Participants

#### Setting

The study will be conducted in the research and clinical centers, School of Chinese Medicine, Hong Kong Baptist University (HKBU). All participants will be recruited from the general public via daily outpatient services and advertisements in newspapers and on a website.

#### Inclusion criteria

Patients who have intermittent or persistent AR, who are 18 years of age or older, regardless of gender, race or educational and economic status will be included. The diagnostic criteria for AR for inclusion will be as follows according to the ARIA criteria [19]: (1) positive skin prick tests, (2) high circulating levels of allergen-specific IgE antibody detected by a specific blood test for allergy called a radioallergosorbent (RAST) test, (3) clinical history or identified allergen, (4) nasal provocation (test). The lowest score of the Total Nasal Symptom Score (TNSS) should be 8 or above.

#### Exclusion criteria

Exclusion criteria will be allergic asthma, moderate to severe atopic dermatitis, any autoimmune disorder, any severe chronic inflammatory disease, history of anaphylactic reactions and hypersensitivity to cetirizine or related drugs, specific immunotherapy during the past 3 years or planned in the next 2 years, and pregnancy or breastfeeding.

### Interventions

#### Medications

Participants in the TJ group will be treated with herbal patches on five acupoints on the back. The formula of the herbal patch consists of *Bai Jie Zi* (*Sinapis semen*), *Yan Hu Suo* (*Corydulis rhizoma*), *Zhi Gan Sui* (*Kansui radix*), *Xi Xin* (*Asari radix et rhizama*) and *Rengong She Xiang* (*Moschus artifactus*). The first four of these herbs will be ground into a powder, mixed thoroughly with 20 % *Bai Jie Zi* (*Sinapis semen*), 25 % *Yan Hu Suo* (*Corydulis rhizoma*), 15 % *Zhi Gan Sui* (*Kansui radix*), and 40 % *Xi Xin* (*Asari radix et rhizama*), and the resulting powder will then be mixed with fresh ginger juice in a ratio of 20 g to 25 ml. The mixture will be made in to a 1 cm^2^ round patch weighing 2 g. Artificial *Rengong She Xiang* (*Moschus artifactus*), 0.02 g, will then be placed on top of each patch. Participants in the placebo-control group will be treated with placebo patches on the same acupoints as the TJ group. The placebo patch consists of flour and edible pigments. These two ingredients will be mixed with water and made in to 1 cm^2^ round patches weighing 2 g. Participants in the waitlist-control group will wait for 4 weeks and will not receive any treatment material. The composition and the action of each herb in the TJ herbal patches are listed in Table 1.

**Table 1:** Composition and action of Tian Jiu (TJ) herbal patches

Ingredients	Percent	Action
*Sinapsis semen*	8.79	TCM: 1. To warm the lung and sweep phlegm, 2. To disinhibit *qi*, 3. To dissipate binds and unblock collaterals to relieve pain
		Pharmaceutical study: 1. Antibacterial effect, 2. Skin stimulating effect, 3. Effect on gastric secretion, 4. Emetic effect, 5. Regulation of blood pressure, 6. Anti-lipid peroxidation, 7. Expectorant effect
*Corydulis rhizoma*	11.01	TCM: 1. To activate blood, 2. To move *qi,* 3. To relieve pain
		Pharmaceutical study: 1. Effect on the CNS (analgesic effect, hypnotic effect, sedative and tranquilizing effects), 2. Muscular relaxant effect, 3. Cardiovascular effect, 4. Effect on gastric secretion and on urination, 5. Effect on pituitary-adrenocortical function
*Kansui radix*	6.56	TCM: 1. To expel retained fluid by purgation, 2. To disperse swelling and dissipate binds
		Pharmaceutical study: 1. Purgative effect
*Asari radix et rhizama*	17.69	TCM: 1. To dispel wind and dissipate cold, 2. to dispel wind and relieve pain, 3. To open the orifices, 4. To warm the lung and resolve fluid retention
		Pharmaceutical study: 1. Sedative and analgesic effects, 2. Antipyretic and anti-inflammatory effects, 3. Respiratory effect, 4. Cardiovascular effect, 5. Anti-histaminic and anti-allergic effects
*Moschus Artifactus*	0.45	TCM: 1. Induce resuscitation and restore consciousness, 2. Promote blood circulation to remove obstruction, 3. Alleviate pain
		Pharmaceutical study: 1. Effects on the CNS, 2. cardiovascular effects, 3. Anti-inflammatory effect, 4. Effects on the uterus
*Zingiberis rhizoma recens* (fresh ginger juice)	55.5	TCM: 1. To release the exterior and dissipate cold, 2. To warm the middle energizer to check vomiting, 3. To resolve phlegm and suppress cough, 4. To reduce toxin of fishery product
		Pharmaceutical study: 1. Effects on the digestive system, 2. Anti-emetic effect, 3. Effect on the CNS

*CNS* central nervous system *TCM* traditional Chinese medicine

#### Acupoints

The choice of acupoints was based on a literature review and clinical practice. A survey conducted in 2010 by the School of Chinese Medicine at HKBU revealed that 60 % of patients who were suffering from AR or asthma found their symptoms were alleviated after receiving TJ therapy. Amongst the 812 people receiving TJ therapy, 558 (approximately 70 %) were suffering from AR or asthma. The survey covered 224 AR patients and 57 asthma patients, and found that 60 % of each group reported that their symptoms were relieved [20]. The latest clinical study conducted in 2014 revealed that more than 50 % of patients suffering from AR obtained benefit after receiving TJ therapy. The study found that, among the 3438 patients included in the survey, 52–67 % of the patients with AR found that their symptoms were alleviated and 32–50 % of those surveyed reported that their body constitution had been improved [21].

Five acupoints will be used: *Dazhui* (GV 14), *Feishu* (UB 13) and *Shenshu* (UB 23), on both sides of the body. A TJ patch will be applied to each acupoint, and left on for 2 hours. A 4 cm^2^ piece of hypoallergenic tape will be used to stick one patch on one acupoint. After 2 hours the patches will be removed by our Chinese medicine practitioner (CMP). If a participant cannot tolerate the stimulation or suffers a allergic reaction, the patching time can be appropriately shortened. Names and details of acupoints are listed in Table 2.

**Table 2:** Acupoints used in the study

Name of acupoints	Areas	Special qualification	Effects of stimulation	Indications
*Dazhui* (GV 14)	In a depression below the processus spinosus of the 7th cervical and above the 1st thoracic vertebrae	A crossing point by which the leading sinartery communicates with all *yang* conduits	Stabilising and regulating the *qi* of centre; strengthening the hepatic and pulmonal orb, keeping the nasal passages open	Cough and asthma
*Feishu* (UB 13)	1.5 *cun* laterally of the processus spinosus of the 3rd thoracic vertebrae	Dorsal inductor (inductorium dorsale) for the pulmonal orb	Stabilising and harmonizing the pulmonal and the renal orb; regulating the *qi*; strengthening the *yin*	Cough with asthma, common cold, nasal congestion
*Shenshu* (UB 23)	1.5 *cun* laterally of the 2nd lumbar vertebrae	Dorsal inductor of the renal orb	Strengthening and harmonizing the renal orb; improving hearing and clearing vision	Cough, asthma, asthenic breathing

### Outcomes

The primary outcome will be the change in the weekly average of the Total Nasal Symptom Score (TNSS) recorded in participants’ diaries from baseline to the end of treatment [22]. The TNSS consists of four nasal symptoms (rhinorrhea, nasal itching, nasal obstruction, and sneezing) using a five-point scale from 0 to 4 (0 = no symptom, 1 = mild, 2 = moderate, 3 = severe, 4 = very severe). The TNSS will be obtained from the sum of all four individual symptom scores, with a total possible score ranging from 0 (no symptoms) to 16 (maximum symptom intensity). This scoring system has been validated [23, 24].

The secondary outcomes will be changes in symptoms and changes in need for medication between baseline and the end of treatment. Symptoms will be assessed by using the RQLQ, which has 28 questions in seven domains (activity limitation, sleep problems, nasal symptoms, ocular symptoms, other symptoms, practical problems, and emotional function) ranked from 0 (no impairment) to 6 (severe impairment) [25]. Rescue medication need will be measured using an RM score (RMS), comprising the weekly sum of daily assessments. Rescue medicine usage will be scored daily on a four-point scale (0 = no rhinitis medicine; 1 = cetirizine, 10 mg/d, or equivalent; 2 = cetirizine, 20 mg/d, or equivalent; 3 = systemic or topical corticosteroids for AR) (daily range; 0 to 3; weekly range: 0 to 21). If more than one RM is used on the same day, only the maximal score medication will be recorded [26].

For safety concern, we will record every adverse event during the treatment and follow-up, and make statistical comparisons. Treatment will be suspended immediately should serious adverse events occur. Further assessment will be needed to decide whether the trial should be suspended.

### Randomization assignment

This study is designed as a randomized, waitlist-control trial. Eligible participants will be randomly and equally allocated to one of the three groups: T (TJ) group, P (placebo) group, and W (waitlist-control) group. Random Allocation Software (version 1.0.0, Isfahan, Iran) will be used to generate a randomization scheme. The principal investigator (PI) will generate the random allocation sequence, and will not share it with other investigators. After the run-in stage, a research assistant (RA) will assign treatments according to the codes which are kept in opaque sealed envelopes with consecutive randomization numbers. Emergency unblinding can only happen when the intervention information is compulsory for the participant’s future management.

### Sample size

Sample size calculations are based on the primary outcome measurement. Since this is a pilot clinical study, the improvement rate of group W would obviously be 0. Subsequently, we assume that there will be 30 % improvement of TNSS symptoms in group T and 15 % in group P. Considering a power of 80 %, and a alpha value of 2.5 % (two-tailed), the sample size will be calculated using the following formula:

$$ n=\frac{2\lambda }{{\left(2{ \sin}^{-1}\sqrt{P_t}-2{ \sin}^{-1}\sqrt{P_w}\right)}^2}, $$

The λ is the cut-off value. *Pt* is 30 % and *Pw* is 0. Then each group will need at least 38 subjects for significance to be detected [27]. Allowing for a 20 % dropout, we plan to recruit 46 subjects in each group, making a total of 138.

### Data processing and analysis

All efficacy and safety analyses will be conducted according to the intention-to-treat (ITT) principle. Missing values will be imputed by the last-observation-carried-forward method. The statistical analysis will be performed using the Statistical Packages for the Social Sciences (SPSS) for Windows, version 21.0. Statistical significance is defined as a two-sided *P* value of <0.05. Baseline characteristics will be reported as mean (SD). Comparisons between groups will be conducted by using an analysis of covariance (ANCOVA) with baseline as covariate. All items and subscales will be compared between groups for each 4-week period using ANCOVA, with treatment group as a factor in the model and baseline as the covariate. The changes in scores from baseline to the end-point of treatment will be tested using repeated measure analysis of variance (ANOVA). Within-group differences will be assessed using a paired *t* test for normally distributed data and a Wilcoxon signed-rank test for non-normally distributed data.

### Data collection and handling of withdrawal and dropout

This is a 9-week clinical trial, in which subjects must take TJ for 8 weeks, attend five assessment visits, complete a set of questionnaires, and stop taking other medications for symptom control. All collected information will be input into a computer by a RA. In the meantime, all the files will be stored in numerical order. Participant files will keep in storage for 5 years.

Considering maximization of participants’ compliance, we will firstly run a thorough consent process for all subjects by interpreting the details of the trial schedule, potential adverse events, and the responsibilities of researchers and participants. In addition, before every visit, a RA will contact the subject to re-confirm the schedule. If a participant shows signs of wanting to withdraw, we would try to understand the possible reasons and aim to find the best option for encouraging the participant to remain in the study.

## Discussion

TJ, also known as natural moxibustion is a conventional method of treating respiratory allergic diseases in TCM. The procedure is convenient and inexpensive. Thousands of people accept this treatment in China every year. Laboratory study has found that natural moxibustion treatment can reduce IgE and interleukin (IL)-4 levels in the plasma of asthmatic rats, as well as airway inflammation [28]. Clinical studies have found that TJ can modulate anti-inflammatory cytokines [17], and that it appears to work by neuro-immunological mechanisms. However, there have been no methodologically controlled clinical studies confirming the efficacy of TJ therapy.

Therefore, our team has designed this randomized, single-blinded, controlled trial to evaluate the effectiveness of TJ therapy for AR. To our knowledge, this will be the first study to compare TJ therapy with a placebo-control group, as well as a waitlist-control group.

We use pigmented flour mixed with water to make the sham TJ treatment. However, some articles mention that in many studies, sham acupuncture is equally effective as acupuncture [29, 30]. Although the sham TJ does not have the pharmacological effects, the operating procedures still causes a pressing sensation to the skin. Because we use the same acupoints for both groups, pressing these acupoints may create an effect like the acupressure. Therefore, we have designed the study with three groups. One group (group T) will receive traditional TJ therapy, which means the application of herbal patches to specific acupoints on the skin. A second group (group P) will receive sham TJ therapy, which means the application of patches with an inert, similar appearance to the TJ patch on the same acupoints. A third group (group W) will not receive any treatment material in order to examine the influence of sham TJ on the study results.

The trial protocol is the foundation of every clinical study. To facilitate appropriate reference standards for scientific, ethical and safety issues before the trial begins, the protocol of this study has been developed according to Consolidated Standards of Reporting Trials (CONSORT) statement [31], the Standard Protocol Items: Standards for Reporting Interventions in Controlled Trials of Acupuncture (STRICTA) statement 2010 [32], and Standard Protocol Items: Recommendations for Interventional Trials (SPIRIT) statement 2013 [33].

The results of this study are expected to provide consolidated evidence for the effectiveness and safety of TJ for the treatment of patients with AR.

### Trial status

At the time of manuscript submission the study is preparing to enroll participants; no patient has yet begun treatment.

## Abbreviations

AR: allergic rhinitis
ANCOVA: analysis of covariance
CONSORT: Consolidated Standards of Reporting Trials
CMP: Chinese medicine practitioner
ITT: intention-to-treat principle
PI: principal investigator
RA: research assistant
RMS: rescue medication score
RQLQ: Rhinitis Quality of Life Questionnaire
SD: standard deviation
SPIRIT: Standard Protocol Items: Recommendations for Interventional Trials
SPSS: Statistical Packages for the Social Sciences
STRICTA: STandards for Reporting Interventions in Controlled Trials of Acupuncture
TNSS: Total Nasal Symptom Score
